# M-RNA Gene Expression of INF-Γ and IL-10 during Intestinal Phase of *Trichinella spiralis* after Myrrh and Albendazole Treatment

**Published:** 2017

**Authors:** Hanaa Y. BAKIR, Rasha AH ATTIA, Abeer E MAHMOUD, Zedan IBRAHEIM

**Affiliations:** 1. Dept. of Parasitology, Faculty of Medicine, Assiut University, Assiut, Egypt; 2. Dept. of Pharmacognosy, Faculty of Pharmacy, Assiut University, Assiut, Egypt

**Keywords:** *Trichinella spiralis*, Albendazole, Myrrh, IFN-γ, IL-10

## Abstract

**Background:**

The protective response developed against *Trichinella spiralis* infection provokes immune and inflammatory responses mediated by cytokines released from T helper cells. We aimed to evaluate the effect of albendazole or myrrh on the expression of IFN-γ and IL-10 in BALB/c mice infected with *T. spiralis*.

**Methods:**

This study was done at the Animal House of Faculty of Medicine, Assiut University (Assiut, Egypt) from April to December 2015. Mice were infected with 300 *T. spiralis* larvae and treated with albendazole (50 mg/kg per day) or myrrh (500 mg/kg per day) for 3 consecutive days post-infection (pi). The expression of INF-γ and IL-10 was detected in the intestinal tissue by reverse transcription (RT) PCR.

**Results:**

The expression of IFN-γ in mice treated with albendazole and myrrh was detected on days 3 and 15 pi respectively. In the control group, it was found on days 5, 10, 15 and 20 pi with the highest expression on day 15 pi. The expression of IL-10 was detected on days 3, 20 pi in the albendazole and myrrh treated groups, respectively. In the control group, IL-10 expression appeared on days 5 and day 20 pi.

**Conclusion:**

The target of albendazole and myrrh on the profile of IFN-γ and IL-10 on these cytokines were encouraging to reinforce their therapeutic use against trichinellosis.

## Introduction

*T. spiralis* is a worldwide zoonotic parasite. Humans are infected by eating raw or undercooked meat or meat products from infected and imported animals (1). *T. spiralis* initiates infection by penetrating the small intestinal epithelial cells that require rapid indications for the acute inflammatory reaction by the production of pro-inflammatory and inflammatory mediators.

In the acute stages of infection, the inflammation process is characterized by neutrophils and macrophages/monocytes infiltration in the lamina propria. In the late stages of infection, the number of mast cells rises in the lamina propria and between epithelial cells (2). The expulsion of *T. spiralis* adult worms’ from the small intestine is a complex process mediated by immune system. It involves mucosal mast cells and Th2 cells activation with Th1-type cytokine, IFN-γ production during the early stage of infection. Cytokines elaborated by Th1 and Th2 cells stimulate antagonistic types of immunity but other studies suggest that strong responses of Th1 and Th2 develop simultaneously and one response does not necessarily prevent or inhibit the other (3). The regulatory cytokine IL-10 (Th2-type) inhibits the release of pro-inflammatory cytokine and prevents liver necrosis in trichinellosis acute stage (3–5). Improvement of signs of inflammation during experimental trichinellosis is correlated with the increase of regulatory cytokines IL-10, TGF-β and regulatory T cells and down-regulation of pro-inflammatory cytokines; IFN-γ, IL-6, and IL-17; in the spleens, mesenteric lymph nodes and colon of mice (6).

Albendazole is a benzimidazole drug that is used worldwide against a wide variety of human helminthic infections including *Trichinella.* (7–11). The host defense mechanisms can be affected by its stimulatory effect on enzymes and mediators involved in host biochemical defense including glutathione transferase (GST) and inducible nitric oxide synthase (iNOS) respectively (11–13). The albendazole effect on the modulation of T cell immune activation profile and production of Th1/Th2 cytokines during helminthes infection was investigated by many authors on different helminthes (14–18).

Myrrh is an aromatic gum resin resulting from the trunk of *Commiphora molmol* (*C. molmol*) and is used as a traditional natural medicine. It has anti-inflammatory, antimicrobial, anti-bacterial and anti-fungal effects (19). It also has fasciolicidal, molluscicidal, schistosomicidal effects proved effective against intestinal and muscular phases of *T. spiralis* (20), with results nearly comparable to those achieved by albendazole (11). *C. molmol* reduces hepatic injury by down-regulation of inflammatory mediators and cytokines such as TNF-α, IL-6, IL-10 and iNOS. This property might be sufficient to combat cellular damage and could be a potential clinical application (21). Five bioactive compounds derived from myrrh and frankincense (an aromatic resin belongs to genus Boswellia) could inhibit the expression of IL-1β, IL-2, IL-10, IL-12, TNF-α, INF-γ (22).

We aimed to assess the expression of two different cytokines as a representative for inflammatory and anti-inflammatory cytokines (INF-γ and IL-10) in the intestinal tissues of experimental trichinellosis in mice and treated with albendazole (reference drug) or myrrh (plant extract). Here, we had an opportunity to explore a potential new target of albendazole and myrrh besides their direct deworming effect on reinforcing the therapeutic use of these medications against trichinellosis.

## Materials and Methods

### Parasites and experimental animals

The strain of *T. spiralis* isolated from a naturally infected pig obtained from El-Bassatine Abattoir, Cairo. It was maintained by routine in vivo passages in BALB/c mice. The study was done at the animal house of Faculty of Medicine, Assiut University (Assiut, Egypt) from April to December 2015 under specific pathogen-free conditions. The infected mouse carcasses were skinned and minced then digested in 1% pepsin-hydrochloride and incubated at 37 °C overnight. The isolated infective larvae were washed several times in 0.85% NaCl, after that the larval number per ml was determined (23).

### Study design

Fifty-five BALB/c mice free from parasites (20–25 g) aged 6–8 wk were used. Forty-five of them were divided into three groups (G1, G2, and G3) of 15 mice each. These groups were orally infected with about 300 muscle larvae of *T. spiralis* per mouse; the remaining 10 mice were kept as a non-infected non-treated control group. The first and second group (G1and G2) were treated orally with 50 mg/kg/day albendazole and 500 mg/kg/day myrrh (*C. molmol*), respectively (11), starting from the first day of infection for three successive days while the third group (G3) was the control group (untreated).

### Reference drug

Albendazole suspension 20 mg/ml was from the Egyptian International Pharmaceutical Industries Co.

### Plant material and extract preparation

Myrrh is an oleo-gum-resin (*C. molmol*) which belongs to the Burseraceae family. The extraction of myrrh was done (11, 24). The Cytotoxicity assay (CTAs) on tissue culture cells was done (11, 25). Evaluation of microbial contamination and Bacterial Endotoxin Test (BET) were done (26, 27).

### Tissue sample collection for RNA extraction

In order to study the cytokines profile of IFN-γ and IL-10 over a 20-d period, 3 mice of each group (G1, G2 and G3) and two from the non-infected control group were sacrificed on days 3, 5, 10, 15 and 20 pi (28). At necropsy, 1-cm-long fragments of the small intestine were taken from each mouse starting with 4 cm from the gastro-duodenal junction (29). We washed the samples several times in PBS, preserved in RNA later (RNA stabilization reagent, Imgenex), and stored at 80 °C until used for PCR.

### Extraction of RNA

RNA extraction from homogenized tissues was according to the manufacturer’s instructions QIAamp RNA Blood Mini Kit (Qiagen ®, Germany). The RNA was resuspended in 50 μl of RNase water and by spectrophotometer at an optical density at 260 nm (OD_260_) was quantified.

### Detection of cytokines mRNA by semi-quantitative RT-PCR

To detect mRNAs of IFN-γ and IL-10, total RNA was subjected to reverse transcription using primers of these genes, and such cDNA was further amplified by the PCR method. A cDNA from each sample was prepared using High-Capacity cDNA Reverse Transcription Kits for 200 and 1000 Reactions (Applied Biosystems ®, USA). RT-PCR was achieved on every sample using 1 μg of total RNA (30). Then 5 μl of cDNA were used as a template for PCR amplification, with Go Taq ® G2 Green Master Mix (Promega, USA) in final volume of 30 μl. Forty cycles of amplifications were run under conditions of denaturation at 95 C for 40 sec and extension at 72 C for 1 min. Initial denaturation was carried out for 3 min and the final extension for 10 min. Amplified DNA was analyzed by electrophoresis on 1% agarose gel stained with ethidium bromide. The PCR products were visualized and analyzed using the BioDoc Analyze 2.1 (Biometers ®, Germany). Primers of IFN-γ and IL-10 were as follows: IFN-γ (sense 5′-AAT GAA CGC-TAC ACA CTG CA-3′ and anti-sense 5′-TGA AGA AGG TAG TAA TCA GG-3′) and IL-10 (sense 5′-GGA CAA CAT ACT GCT AAC CGG-3′ and antisense 5′-ATA TTT CGG AGA GAG GTA CA-3′).

### Statistical analysis

The evaluations of the collected data were by Statistical Package for Social Sciences ver.20 for Windows. The values were expressed as mean ± standard deviation. Between the groups, the significance of differences was calculated by ANOVA test. The statistically significant *P-*value was <0.05.

### Ethics

The experimental animal studies were conducted in accordance with the international valid guidelines and were maintained under convenient conditions at the Animal House, Faculty of Medicine, Assiut University.

## Results

### Cytotoxicity assays (CTAs) on tissue culture cells

Myrrh extract achieved the acceptance standards for the CTAs.

### Microbial contamination and Bacterial Endotoxin Test (BET)

Myrrh extract was proved safe and fulfilled the criteria determined by the European Directorate for the quality of Medicines & HealthCare. In addition, BET showed absence of bacterial endotoxins in our herbal product

### Cytokine gene expression after infection with T. spiralis in mice treated with albendazole and myrrh

**Fig. 1:**
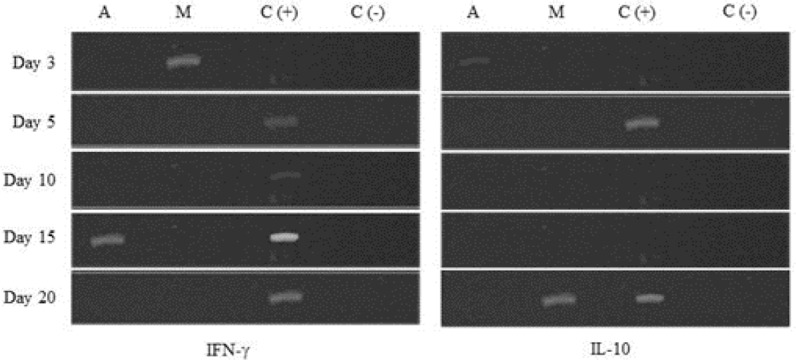
Detection of cytokine mRNAs from intestinal tissues of different mice groups during *T. spiralis* infection; An (Albendazole), M (Myrrh), C+ (Control positive) and C− (Control negative)

To discover the mechanism that influences the modulation of the immune system in mice infected with *T. spiralis* and treated with albendazole and myrrh, we examined the expression of IFN-γ and IL-10. The production of cytokine mRNA during *T. spiralis* infection was compared between the three groups of mice (G1, G2, and G3). The expression of cytokines mRNA of IFN-γ and IL-10 was detected in jejunal tissue on days 3, 5, 10, 15 and 20 by RT-PCR. In the group of mice treated with albendazole (G1), the production of IFN-γ was only seen on day 15 pi. Surprisingly, in the group of mice treated with myhrr (G2), the expression of IFN-γ was detected early on day 3 pi (acute phase). In the control group (G3), the detection of IFN-γ was found on days 5, 10, 15 and 20 pi with a higher expression on day 15 pi (Fig. 1).

Anti-inflammatory cytokine (IL-10) was examined in the previously mentioned mice groups. The results of PCR products of IL-10 were completely different from those found with IFN-γ. The expression of IL-10 in mice appeared early on day 3 pi in (G1) while the group of mice treated with myrrh (G2) showed expression of IL-10 late on day 20 pi (stage of migration). In the control group (G3), IL10 expression appeared on days 5 and day 20 pi (Fig. 1).

We presented our data in a quantitative form by using BioDoc Analyze 2.1 software and measured the band intensity for the cytokine-specific RT-PCR product illustrated in (Table 1). The influence of *T. spiralis* infection on the expression of IFN-γ mRNA showed a significant difference in the level of IFN-γ expression between the albendazole-treated group and the non-infected non-treated group (7.19 ng/μl) on day 15 pi. There was another significant difference between the levels of IFN-γ in non-infected non-treated group among days 5, 10, 15, 20 pi with a significant peak of expression on day 15 pi. In sharp contrast to IL-10 production, it showed nearly equal lower levels between 2.17 ng/μl and 2.62 ng/μl in the three mice groups without a statistical difference but the non-infected non-treated group showed a relatively higher value (2.62 ng/μl) on day 5 pi (Table 1). The levels of these cytokines in the control group (non-infected mice) were undetectable.

**Table 1:** Semi-quantitative PCR analyze of band intensity showing values of the expression of IFN-γ, and IL-10

	**Albendazole (mean ±SE)**		**Myrrh (mean ±SE)**		**Non-infected non-treated (mean±SE)**	
	**IFN**	**IL-10**	**IFN**	**IL-10**	**IFN**	**IL-10**
Day 3	-	2.17±0.12	3.91±0.09	-	-	-
Day 5	-	-	-	-	3.11±0.15^A^	2.62±0.11
Day 10	-	-	-	2.48±0.11	2.00±0.05^AB^	-
Day 15	4.14±0.13^a^	-	-	-	7.19±0.24^aABC^	-
Day 20	-	-	-	-	5.04±0.17^ABC^	2.58±0.09

The same small letter is significant difference in the same row

The same capital letter is significant difference in the same column

## Discussion

Our herbal extract microbial limit was within the acceptable range that does not cause health hazards to the planned groups or reduces herbal stability (31). The outcome of infection with intestinal nematodes has contributed to the capacity of the intestine to mount immune and inflammatory responses to infection especially those mediated by T cells. The inflammatory processes that accompany these responses may play a vital role in host defense, but can also have pathological concerns (2). The intestinal mucosa and associated lymphoid tissues are the first point of host contact during *T. spiralis* infection (32), and the mechanism of the intestinal expulsion of *T. spiralis* adult worm is known to be dependent on a Th2-type cytokines (IL-4, IL-13, IL-9, and IL-10) which lead to mucosal mast cells activation (3, 33).

The effectiveness of myrrh extract against *T. spiralis* both phases (intestinal and muscular), and its results were nearly similar to albendazole (11). Thus, we focused our work on the recognition of two different cytokines mRNA (IFN-γ and IL-10) produced from intestinal tissue to analyze the modulation of immune response during the *T. spiralis* intestinal phase after treatment with albendazole or myrrh on the indicated days.

In the present study, the expression of IFN-γ was astonishing in the group of mice treated with myrrh (G2) as it showed early expression (Day 3 pi) before the other two groups, which may alarm the immune system about the inflammatory process and enable the host to control larvae production. IFN-γ is involved in protection against newborn larvae and it does not affect the adult worms’ expulsion. Its mechanisms of action against newly born larvae are by enhancement of the cytotoxic killing by granulocytes, eosinophils and activated macrophages (3).

The production of IFN-γ in the mice treated with albendazole (G1) was remarkably delayed until day 15 pi, which indicated that such chemical compound might interfere with early production of this inflammatory cytokine. We were not surprised to find that the expression of IFN-γ in the infected non-treated group (G3) was detected throughout the experimental days (days 5, 10, 15 and 20 pi). The inflammatory process proceeded late in G3 until day 20 pi, which suggested the inability of immune system to combat infection. These results were consistent with previous report showed the early production of IFN-γ from the intestinal tissue of pig infected with *T. spirals* (28).

In the current study, the pattern of anti-inflammatory cytokine gene expression mRNA (IL-10) during *T. spirals* infection was examined in the previous groups of mice. Our data showed amazing results as the production of IL-10 was delayed in the mice treated with myrrh (day 20 pi), in sharp contrast to its early expression in the mice treated with albendazole (day 3 pi). The delayed production of IL-10 until day 20 pi in the mice treated with myrrh permits IFN-γ to employ its defensive effects against newly born larvae that in turn may restrict the number of larvae entering blood circulation (3). However, in the mice treated with albendazole, the expression of IL-10 was early (day 3 pi) even before the expression of IFN-γ, and the host did not get the benefit of the balancing effect of anti-inflammatory cytokine. Meanwhile, IL-10 is essential for *T. spiralis* adult worms’ expulsion from the small intestine via Th1 and Th2 responses regulation at mucosal surfaces (3). In addition, it regulates the protective host immune response against the newly born larvae (34, 35). Nevertheless, the expression of IL-10 in the infected non-treated control group of mice was produced during early and late stage (day 5 and day 20 pi) of infection. Our observations about the importance of IL-10 are in harmony with a prior work (4, 5), which revealed the essential role of IL-10 in preventing the migration of intestinal T cells to the liver and inhibiting the development of hepatitis in mice infected with *T. spirals*. The balance between IFN-γ and IL-10 is important to determine the immunity against various stages of the parasite (3).

As far as we know, no available published literature detected the effect of albendazole and myrrh treatment on gene expression of cytokines mRNA during experimental trichinellosis in mice. However, the combined use of myrrh and frankincense (an aromatic resin) was significant in suppressing arthritis progression mainly by reduction of pro-inflammatory factors (22). While in other study, myrrh extracts showed high iNOS (biochemical inflammatory mediator) expression during trichinellosis in experimental animal model, which is nearly similar to albendazole, and this high expression may efficiently modify the defense mechanisms of the host (11).

The effect of albendazole treatment either alone or combined with other medications on the Th1 and Th2 plasma cytokines during other helminthes infection was evaluated. Albendazole treatment during *Ascaris lumbricoides* infection might decrease worm load leading to a reduction in IL-10 production, which ultimately improved cellular immunity and CD4^+^T cell counts (17). The combined therapy of albendazole and astragalus (plant belonging to family Fabaceae used for immune enhancement) adjust the Th1 / Th2 cytokine levels which decrease the development and progression of liver injury caused by *clonorchis sinensis* infection (18).

## Conclusion

Treatment with albendazole and myrrh were exhibited different cytokine patterns. Their effect on the cytokine profile of INF-γ and IL-10 is promising to reinforce their therapeutic use against trichinellosis. Nevertheless, further characterization will be needed at the cellular level to identify the cells and other cytokines involved in trichinellosis.

## References

[1] Morales MA Mele R Sanchez M Sacchini D De Giacomo M Pozio E Increased CD8^+^T cell expression and a type 2 cytokine pattern during the muscular phase of *Trichinella* Infection in humans. Infect Immun. 2002; 70: 233– 9. 11748188 10.1128/IAI.70.1.233-239.2002 PMC127601

[2] Li CKF Seth R Gray T Bayston R Mahida YR Wakelin D Production of proinflammatory cytokines and inflammatory mediators in human intestinal epithelial cells after invasion by *Trichinella spiralis*. Infect Immun. 1998; 66 (5): 2200– 6. 9573108 10.1128/iai.66.5.2200-2206.1998 PMC108182

[3] Helmby H Grencis RK Contrasting roles for IL-10 in protective immunity to different life cycle stages of intestinal nematode parasites. Eur J Immunol. 2003; 33 (9): 2382– 90. 12938214 10.1002/eji.200324082

[4] Bliss SK Alcaraz A Appleton JA IL-10 prevents liver necrosis during murine infection with *Trichinella spiralis*. J Immunol. 2003; 171 (6): 3142– 7. 12960341 10.4049/jimmunol.171.6.3142

[5] Bliss SK Bliss SP Beiting DP Alcaraz A Appleton JA IL-10 regulates movement of intestinally derived CD4^+^ T cells to the liver. J Immunol. 2007; 178 ( 12) 7974– 7983. 17548634 10.4049/jimmunol.178.12.7974

[6] Yang Y Wang Y Zhan B Gu Y Cheng Y Zhu X Excretory/secretory products from *Trichinella spiralis* adult worms ameliorate DSS-induced colitis in mice. PLos One. 2014; 9 (5): e96454. 24788117 10.1371/journal.pone.0096454 PMC4008629

[7] Chung MS Joo KH Quan FS Kwon HS Cho SW Efficacy of flubendazole and albendazole against *Trichinella spiralis* in mice. Parasite. 2001; 8 (2 Suppl): S195– 8. 11484354 10.1051/parasite/200108s2195

[8] Siriyasatien P Yingyourd P Nuchprayoon S Efficacy of albendazole against early and late stage of *Trichinella spiralis* infection in mice. J Med Assoc Thai. 2003; 86 Suppl 2 : S257– 62. 12929998

[9] Shoheib ZS Shamloula MM Abdin AA El-Segai O Role of α-chymotrypsin and colchicine as adjuvant therapy in experimental muscular trichinellosis: parasitological, biochemical and immunohistochemical study. Egypt J Med Microbiol. 2006; 15: 773– 790.

[10] Shalaby MA Moghazy FM Shalaby HA Nasr SM Effect of methanolic extract of *Balanites aegyptiaca* fruits on enteral and parenteral stages of *Trichinella spiralis* in rats. Parasitol Res. 2010; 107: 17– 25. 20349194 10.1007/s00436-010-1827-9

[11] Attia RAH Mahmoud AE Farrag HM Makboul R Mohamed ME Ibraheim Z Effect of myrrh and thyme on *Trichinella spiralis* enteral and parenteral phases with inducible nitric oxide expression in mice. Mem Inst Oswaldo Cruz. 2015; 110: 1035– 1041. 26676322 10.1590/0074-02760150295 PMC4708024

[12] Zeromski J Boczoń K Wandurska-Nowak E Mozer-Lisewska I Effect of aminoguanidine and albendazole on inducible nitric oxide synthase (iNOS) activity in *T. spiralis*-infected mice muscles. Folia Histochem Cytobiol. 2005; 43: 157– 9. 16201316

[13] Wojtkowiak-Giera A Wandurska-Nowak E Michalak M Derda M Lopaciuch J Trichinellosis in mice: effect of albendazole on the glutathione transferase in the intestines. Folia Parasitol (Praha). 2012; 59: 311– 4. 23327014 10.14411/fp.2012.044

[14] Chen KM Lai SC Biochemical and pathological evaluation of albendazole/thalidomide co-therapy against eosinophilic meningitis or eningoencephalitis induced by *Angiostrongylus cantonensis*. J Antimicrob Chemother. 2007; 59: 264– 276. 17298991 10.1093/jac/dkl492

[15] Cooper PJ Moncayo AL Guadalupe I Benitez S Vaca M Chico M Griffin GE Repeated treatments with albendazole enhance Th2 responses to *Ascaris lumbricoides* but not aeroallergens in children from rural communities in the Tropics. J Infect Dis. 2008; 198: 1237– 1242. 18729781 10.1086/591945 PMC2635537

[16] Diao Z Chen X Yin C Wang J Qi H Ji A *Angiostrongylus cantonensis*: effect of combination therapy with albendazole and dexamethasone on Th cytokine gene expression in PBMC from patient with eosinophilic meningitis. Exp Parasitol. 2009; 123: 1– 5. 19559022 10.1016/j.exppara.2009.04.016

[17] Blish CA Sangaré L Herrin BR Richardson BA John-Stewart G Walson JL Changes in plasma cytokines following treatment of *Ascaris lumbricoides* in HIV-1 infected individuals. J Infect Dis. 2010; 201: 1816– 1821. 20441516 10.1086/652784 PMC2946624

[18] Jin-Fu Y Jie Y Yuan L Influence of albendazole combined with astragalus injection on Th1/Th2 cytokines of patients infected with *Clonorchis sinensis*. Chinese General Practice. 2013; 33.

[19] Dolara P Corte B Ghelardini C Pugliese AM Cerbai E Menichetti S Lo Nostro A Local anaesthetic and antifungal properties of sesquiterpenes from myrrh. Planta Med. 2000; 66: 356– 358. 10865454 10.1055/s-2000-8532

[20] Basyoni MM El-Sabaa AA Therapeutic potential of myrrh and ivermectin against experimental *Trichinella spiralis* infection in mice. Korean J Parasitol. 2013; 51: 297– 304. 23864740 10.3347/kjp.2013.51.3.297 PMC3712103

[21] Ahmad A Raish M Ganaie MA Hepatoprotective effect of *Commiphora myrrha* against d-GalN/LPS-induced hepatic injury in a rat model through attenuation of pro-inflammatory cytokines and related genes. Pharm Biol. 2015; 53: 1759– 67. 25864920 10.3109/13880209.2015.1005754

[22] Su S Duan J Chen T Corrigendum: Frankincense and myrrh suppress inflammation via regulation of the metabolic profiling and the MAPK signaling pathway. Sci Rep. 2015; 5: 15597. 26513369 10.1038/srep15597 PMC4625473

[23] Gamble HR Detection of trichinellosis in pigs by artificial digestion and enzyme immunoassay. J Food Prot. 1996; 59: 295– 8. 10463449 10.4315/0362-028x-59.3.295

[24] Evans WC Trease, Evans’ Pharmacognosy, 15th ed., Elsevier, Edinburgh, 2002; 585.

[25] Repetto G Del Peso A Zurita JL Neutral red uptake assay for the estimation of cell viability/cytotoxicity. Nat Protoc. 2008; 3: 1125– 1131. 18600217 10.1038/nprot.2008.75

[26] EDQM Council of Europe 2014 European Pharmacopoeia. edqm.eu/en/european-pharmacopoeia-8th-edition-1563.html .

[27] Hussaini SN Hassanali HT Limulus amoebocyte lysate assay of endotoxin: a method for visual detection of the positive gel reaction. J Med Microbiol. 1987; 24: 89– 90. 3612746 10.1099/00222615-24-1-89

[28] Picherot M Oswald IP Cote M Swine infection with *Trichinella spiralis*: Comparative analysis of the mucosal intestinal and systemic immune responses. Vet Parasitol. 2007; 143: 122– 30. 16962244 10.1016/j.vetpar.2006.08.003

[29] Despommier DD *Trichinella spiralis* and the concept of niche. J Parasitol. 1993; 79: 472– 482. 8331468

[30] Dozois CM Oswald E Gautier N A reverse transcription-polymerase chain reaction method to analyze porcine cytokine gene expression. Vet Immunol Immunopathol. 1997; 58: 287– 300. 9436272 10.1016/s0165-2427(97)00039-1

[31] Moreira DL Teixeira SS Monteiro MH Traditional use and safety of herbal medicines. Rev Bras Farmacogn. 2014; 24: 248– 257.

[32] Christ AD Blumberg RS The intestinal epithelial cells: immunological aspects. Springer Semin Immunopathol. 1997; 18: 449– 461. 9144864 10.1007/BF00824052

[33] Khan WI Vallance BA Blennerhassett PA Critical role for signal transducer and activator of transcription factor 6 in mediating intestinal muscle hypercontractility and worm expulsion in *Trichinella spiralis*-infected mice. Infect Immun. 2001; 69: 838– 44. 11159976 10.1128/IAI.69.2.838-844.2001 PMC97960

[34] Kang SA Cho MK Park MK Alteration of helper T-cell related cytokine production in splenocytes during *Trichinella spiralis* infection. Vet Parasitol. 2012; 186: 319– 27. 22222009 10.1016/j.vetpar.2011.12.002

[35] Kim S Park MK Yu HS Toll-Like receptor gene expression during *Trichinella spiralis* infection. Korean J Parasitol. 2015; 53: 431– 8. 26323841 10.3347/kjp.2015.53.4.431 PMC4566501

